# The “Hotel Endemic” at Washington

**Published:** 1857-09

**Authors:** Robert King Stone, James E. Morgan

**Affiliations:** Com. Board Health.; Washington; Com. Board Health.; Washington


From the Virginia Medical Journal.

### *The "Hotel Endemic" at Washington.*

We have refrained from any remarks upon the sickness which prevailed at the National Hotel in Washington, during January and February last, while without other data respecting it than the vague and unauthenticated statements of newspapers. The official investigations, which have since been instituted, enable us to place on record the plain facts of the case. These, as they were elicited by a committee on the board of health, are concisely stated in a letter, from the pen of the professor of anatomy in the Washington Medical College.

The greater number of those persons who were attacked with the "hotel sickness," were attended by Drs. Boyle and Hall. We reproduce in full the testimony of the latter before the board of health. The opinion of its learned, sagacious, and experienced author will have great weight with medical men, though it may not satisfy an excited public sentiment, which prefers to view the hotel calamity as a new Guy Fawke's plot, rather than a solemn lesson that hygienic laws cannot be violated with impunity:

*Testimony of Dr. James C. Hall.*—I am a practising physician in the city of Washington. I saw one of the first cases of the disease that recently made its appearance in the National Hotel. The patient occupied a room in the fourth story, and was affected with diarrhœa, loss of appetite, and irritable stomach. This was in the latter part of January. About the first of February I was called to see other cases, the weather at that time being extremely cold. Usually the disease would make its first appearance in the morning, the patient having retired at night apparently well. Profuse operations, always presenting the same appearance, * * * and of a frothy or yeasty appearance, accompanied with much flatus. If the diarrhœa were checked by opiates or astringents, vomiting supervened, and if the vomiting ceased the purging returned. There was some pain, but not that characteristic of inflammation; rather that caused by distension from flatus. In some protracted cases there was pain upon pressure, but not the pain of acute inflammation. There was a great thirst and a desire for acid drinks. Sometimes there were cramps, but no enteritis. Farinaceous diet was the best. The stools did not present the appearance of metallic poisoning. I have seen repeated attacks of dysentery and cholera in houses, attributed to filth, but they did not present the same appearances as this disease.

I do not believe that the disease at the National Hotel was caused by metallic poisoning. Poisoning by a metallic substance acts as a local irritant, causing pain, tormina, tenesmus * * * * not a profuse diarrhœa, and the disease proceeds steadily either to terminate favorably or fatally. I believe that the cause of the disease that has prevailed at the National Hotel is a blood poison taken into the system through the medium of the lungs; that is, a miasm pervading the atmosphere of the hotel. It may possibly also have been absorbed by the food from the atmosphere. I think that closing the sewer at the corner of Pennsylvania avenue and Sixth street causes the noxious air contained in the sewer to pass into the hotel and disseminate itself throughout the whole house. The heating apparatus is so constructed as to assist in disseminating the vitiated air of the cellars throughout the house. The room immediately above the heater is particularly offensive. There is a flight of stairs coming directly up from the wash room, and the steam and vapor arising therein cause a current of heated air to ascend the stairway, which also causes the effluvia to be distributed throughout the house. During the mild weather in February, the disease entirely ceased for two weeks, the house being well ventilated by the windows remaining open; but when the cold weather returned and the house was closely shut, the disease again made its appearance more violently than before. I drank freely of the water used in the hotel, and it produced no disagreeable effects. I have seen a person who frequented the barber's shop connected with the hotel, although not a boarder in the house, attacked with the disease ; and persons who frequented the house have been affected in the same manner as the boarders, but the disease was not as violent. Persons remaining in the house after having become ill were no worse than those who removed from the house after having been attacked. I attribute the disease to a poisonous miasm. I have had no case that terminated fatally * * * * * I have had altogether thirty or forty cases. I have no patient in the house at the present time. The disease usually returns after being checked for a few days, and these relapses are frequent. I have seen persons accidentally or purposely poisoned by corrosive sublimate, acetate of copper, arsenic, lead, and oxalic acid, and also by arsenic administered as a medicine, but in no case have the symptoms resembled those of the diarrhœa now under consideration.

I beg leave to append the following quotation from Christison on Poisons, Philadelphia edition, page 621 :

"In August, 1831, twenty-two boys living at a boarding school at Clapham were seized in the course of three or four hours with alarming symptoms of violent irritation of the stomach and bowels, subsultus of the muscles of the arms, and excessive prostration of strength. * * * A suspicion of accidental poisoning having naturally arisen, the various utensils and articles of food used by the family were examined without success ; and the only circumstances which appeared to explain the accident was that two days before the first child took ill a foul cess-pool had been opened, and the materials diffused over a garden adjoining to the children's playground. This was considered a sufficient cause of the disease by Dr. Spurgin and Messrs. Angers and Saunders, of Clatham, as well as by Drs. Latham and Chambers and Mr. Pearson, of London, who personally examined the whole particulars. Their explanation may be the only rational account that can be given of the matter. But, as no detail of their chemical enquiries was ever published, their opinion cannot be received with confidence by the medical jurist and physician, since it is not supported, so far as I am aware, by any previous account of the effect of hydro-sulphuric acid gas."

Christison also says, at page 491, "Another affection, sometimes brought on by putrid exhalations, is violent diarrhœa or dysentery ;" and refers to the case of a well known French physician, who from such exposure, was seized with giddiness, nausea, tendency to vomit, violent colic, with profuse diarrhœa.

At a meeting of the New York academy of medicine, May 6th, Dr. Wynne, of Baltimore, being introduced by Dr. Valentine Mott, detailed the facts of the hotel endemic, and referred its cause to the effluvia arising from the ill constructed sewer. He cited several examples of identical diseases produced by similar noxious exhalations.

He stated that Dr. C. T. Jackson, of Boston, who was at Washington, during the prevalence of the disease, very justly remarks that "no chemical or reliable medical evidence has yet been adduced to prove that any one of the persons who were sick with the disease had taken poison of any kind into their stomachs." Now the question arises, can disease, presenting the characteristics of the one just described, be produced by putrid exhalation arising from deficient ventilation? If so, without the adduction of new evidence, the endemic must be attributed to this cause.

Doctor, afterwards Sir John Pringle, the president of the Royal society, than whom no man of this day was a more acute observer, in his observations on the diseases of the English troops in Flanders, says that whenever the marsh, near which the army was stationed, was foul with animal impurities, the soldiers were invariably seized with bowel irritation, amounting even to dysentery. This observation, made by this distinguished army surgeon, has been corroborated by the experience of every one having the medical care of bodies of garrisoned or field troops since his day. The experience of our own army in Florida, and more recently in Mexico, shows the great prevalence and malignity of bowel affections among those who are subjected to putrid exhalations. Nor is this confined to those who are confined to the wretched quarters of the soldiers of an army in the time of war, or to the ill ventilated apartments of the more wretched in populous towns, but often invades the luxurious dwellings of the more opulent classes.

Dr. Rigby, in his evidence before the health of towns commission, says that he has often been enabled to detect, by the sense of smell, the poisonous exhalations from sewers in the more fashionable parts of London. He considers the sense of smell as very important to a physician. A crafty nurse, says he, may hide much from the eye, but she can conceal but little from the nose of a medical man who is at all experienced in these matters. He is clear in attributing an attack of puerperal fever which seized the inmates of the Lying-in hospital under his charge, to defective sewerage and ventilation. Dr. Dray, of Kirk's college, is equally positive in tracing consumption to the same cause, and Dr. Southwood Smith bears ample testimony of its power to produce fever.

There are instances in which the attacks from this cause assume each one or the other of these forms, and others in which two or more are conjoined. This was especially the case in the Croydon epidemic which occurred in 1852. Mr. Granger, who was sent by the board of health to investigate the cause of the outbreak, and who, among other like causes, attributed the epidemic to the effluvia which escaped from the gully-holes of the old sewers, says: Besides the attacks of fever, there was a large amount of diarrhœa. Mr. Thompson had fifty cases in his practice, all evidently attributable to and forming a part of the epidemic. In the Croydon epidemic, a leading characteristic of all the cases of fevers was diarrhœa, and Dr. Granger says that, in this outbreak, gastric disturbance, traceable to putrid effluvia, was uniformly present.

We conclude this subject, for the present, by publishing a circular of the committee of the Washington board of health. It is to be hoped that it will receive an adequate response from those physicians on whose authority the hotel sickness has been ascribed to "metallic poisoning."

The undersigned have been appointed a committee by the board of health of Washington to communicate with residents of this city and persons elsewhere, in order to procure, if possible, further authenticated information in regard to the malady which recently prevailed at, and was known as the "endemic of the National Hotel." No fatal cases have occurred in this community; but, as vague and unauthenticated statements have reached us that persons have died elsewhere from this cause, we take this as the only sure mode of calling upon physicians and persons abroad to favor us with statements of the names and residences of the persons who have thus died. We desire to obtain a description of the course and symptoms of their disease; the opinion formed as to its origin and the reasons therefor; the treatment adopted in the cases, and the results of the post-mortem examinations or chemical analyses of the dejections from the sick, if any such were made. As the guests of that hotel are wide cast over the land, it is impossible for us to obtain the names and residences of their physicians, and must therefore appeal to them, through the public press, for the information desired. Their prompt attention (by letter to the committee) will confer a great favor upon the board of health and this community, and perhaps be of great service in promoting the cause of public health and medical science.

Papers friendly to the above objects will confer a favor on the board of health by publishing this card.

Robert King Stone, M. D.,
James E. Morgan, M. D.
*Com. Board Health.*

Washington, May 7, 1857.

